# Did a workplace sugar-sweetened beverage sales ban reduce anxiety-related sugar-sweetened beverage consumption during the COVID-19 pandemic?

**DOI:** 10.1017/S1368980024000995

**Published:** 2024-05-03

**Authors:** Laurie M Jacobs, Laura A Schmidt, Dean Schillinger, Jamey M Schmidt, Katie E Alegria, Bethany Parrett, Amanda Pickett, Elissa S Epel

**Affiliations:** 1 Philip R Lee Institute for Health Policy Studies, University of California at San Francisco, San Francisco, USA; 2 Department of Humanities and Social Sciences, University of California at San Francisco, San Francisco, USA; 3 UCSF Division of General Internal Medicine, San Francisco General Hospital, San Francisco, USA; 4 UCSF Center for Vulnerable Populations, San Francisco, USA; 5 Sutter Health California Pacific Medical Center Research Institute, San Francisco, USA; 6 Department of Psychiatry and Behavioral Sciences, University of California at San Francisco, San Francisco, USA; 7 Center for Health and Community, University of California at San Francisco, San Francisco, USA

**Keywords:** Sugar-sweetened beverages, Workplace interventions, COVID-19 pandemic, Anxiety

## Abstract

**Objective:**

Workplace sugar-sweetened beverage (SSB) sales bans can reduce SSB consumption. Because stress and anxiety can promote sugar consumption, we examined whether anxiety among hospital employees during the COVID-19 pandemic was associated with changes in SSB consumption and explored whether this relationship varied by exposure to a workplace SSB sales ban.

**Design:**

In a prospective, controlled trial of workplace SSB sales bans, we examined self-reported anxiety (generalised anxiety disorder-7) and self-reported SSB consumption (fluid ounces/d) before (July 2019) and during (May 2020) the COVID-19 pandemic.

**Setting:**

Hospital sites in two conditions (four with SSB sales bans and three without sales bans) in Northern California.

**Participants:**

We sampled 580 participants (hospital employees) from a larger trial of sales bans; all were regular consumers of SSB (minimum 3/week at main trial enrollment). This subsample was chosen based on having appropriately timed data for our study questions.

**Results:**

Across conditions, participants reduced SSB consumption over the study period. However, participants with higher pandemic-era anxiety scores experienced smaller reductions in SSB consumption after 9 months compared with those with lower anxiety scores (*β* = 0·65, *P* < 0·05). When the sample was disaggregated by sales ban condition, this relationship held for participants in the control group (access to SSB at work, *β* = 0·82, *P* < 0·05), but not for those exposed to an SSB sales ban (*β* = 0·42, *P* = 0·25).

**Conclusions:**

SSB sales bans likely reduce SSB consumption through multiple pathways; buffering stress-related consumption may be one mechanism.

Consumption of sugar-sweetened beverages (SSB) is a risk factor for health problems including diabetes, hypertension, CHD, liver disease, abdominal adiposity, gum disease and dental caries. Prior studies have demonstrated the effectiveness of workplace SSB sales bans^(1)^ and their positive impacts on employee health^(2)^. These improvements can also lead to healthcare savings for employers^(3)^.

Our team was conducting a controlled trial of SSB sales bans in hospitals^(1)^ when the COVID-19 pandemic began, creating a natural experiment to test the hypothesis that workplace sales bans might buffer employees from stress-related SSB consumption. Previously, we found that SSB consumption was, on average, decreasing in our sample, with the sales ban reducing consumption even more^(1)^. We anticipated that this downtrend might reverse during the pandemic due to added stress on hospital employees.

Stress is associated with higher intake of high-sugar, ultra-processed, and energy-dense foods and beverages^(4,5)^. Psychological distress is associated with higher consumption of SSB in adults^(6,7)^, and a longitudinal study of adolescents suggested that SSB consumption is used to cope with stress^(8)^. This was demonstrated empirically during the pandemic, when 10 % of adults reported often drinking more SSB than before^(9)^, and people experiencing new financial hardship significantly increased SSB purchases^(10)^. Additionally, higher SSB consumption was cross-sectionally associated with anxiety during the pandemic^(11–13)^. Because of the links between distress and sugar intake, added pandemic-era stress could have influenced SSB consumption in our sample.

Using a subsample from a trial of SSB sales bans^(1)^, we examined anxiety and daily SSB consumption before and during the pandemic. The sample included SSB-drinking employees in hospitals with or without (control) an SSB sales ban. We hypothesised that participants at control hospitals would increase consumption during the pandemic relative to those at hospitals with sales bans, and that differences might be explained by anxiety symptoms.

## Methods

A sales ban removes SSB from workplace sales outlets while still allowing employees to bring SSB purchased elsewhere. The sample for this sub-study was drawn from and extended a larger trial of SSB sales bans^(1)^. When pandemic restrictions halted in-person data collection, we used online methods to observe the impact of pandemic stressors on consumption.

### Participants/Procedures

Participants were employees (*n* 580) at seven Sutter Health hospitals in Northern California (sales ban: *n* 4; non-sales ban control: *n* 3), who reported consuming at least three SSB/week at the start of the main trial. Details of procedures were previously published^(1)^. Participants were included in this sample if they had appropriately timed pre-pandemic data.

Pre-pandemic (T1) data used in this sub-study were collected in 2019 (primarily July), and pandemic-era (T2) assessments were collected in 2020 (primarily May) via online self-report questionnaires. Average time between T1 and T2 was 9 months (271 d), with 81·5 % (*n* 473) sample retention.

### Measures

The primary outcome was change in daily SSB consumption from T1 to T2, measured via the Beverage Intake Questionnaire^(14)^, a validated questionnaire that captures the typical daily quantity-frequency of beverages consumed. SSB included all sugar-sweetened sodas, ‘fruit’ drinks, sports/energy drinks and pre-sweetened coffee/tea drinks. Daily consumption was calculated per beverage by multiplying frequency and quantity, then summed for a total number of fluid ounces per day (oz./d) for each participant. To measure change, we subtracted T2 oz./d from T1 values.

Anxiety was measured at T1 and T2 using the generalised anxiety disorder-7, a commonly used measure of anxiety symptoms^(15)^; participants rated symptoms over the past 2 weeks. This measure had been included pre-pandemic as a potential covariate of consumption.

We collected demographics and measured BMI at the time of recruitment^(1)^.

### Analyses

We fit two sets of regression models where the dependent variable was change in SSB consumption from T1 to T2. First, we focused on anxiety during the pandemic (T2) as a predictor of change in consumption. We fit this model for the full sample and separately for the sales ban and control groups. Coefficients for anxiety in these models can be interpreted as the change in oz./d of SSB consumption per point of anxiety score above the mean. Second, we fit models identical to the first except that the predictor was T1–T2 *change* in anxiety. All regression models were adjusted for sex, race/ethnicity, baseline BMI and T1 consumption.

## Results

The sample was majority female (74·4 %) and ethnically diverse (35·7 % non-Hispanic white; Table 1). A minority of participants (8·0 %) reported switching to remote work at some point during the pandemic; these cases were retained because sensitivity analyses determined that their exclusion did not change the pattern of results. Average anxiety score was 3·0 points (sd 3·8), which corresponds to no/low risk. This increased by 1·2 points (sd 4·1, *P* < 0·001) from T1 to T2, with a larger increase among participants in sales ban hospitals (1·7, sd 4·2) *v*. control hospitals (0·8, sd 3·9, *P* < 0·05; see online supplementary material, Supplemental 1). Because SSB sales bans were already in effect in most ban locations at the start of this sub-study, and because of pre-existing differences, T1 consumption was significantly lower at sales ban hospitals than in control hospitals (22·9 oz./d *v*. 32·9, *P* < 0·001, Table 1). Overall, there was a downtrend in consumption during the 9-month study period, with a mean change of –8·1 (sd 31·7, *P* < 0·001) oz./d (–6·2 sales ban sd 32·1, –9·5 control sd 31·3, *P* = 0·26; see online supplementary material, Supplemental 1). Accordingly, 65·3 % of participants decreased or had no change in consumption. Throughout, consumption and anxiety were positively intercorrelated (*r* = 0·10, *P* < 0·05 at T1; *r* = 0·11, *P* < 0·05 at T2).

**Table 1:** Sample characteristics at T1 (2019)

	All participants (*n* 580)			Control (*n* 324)			Sales Ban (*n* 256)		
	%	Mean	sd	%	Mean	sd	%	Mean	sd
Sex (*n* 579) %									
Female	74·4			79·0			68·6		
Male	25·6			21·0			31·4		
Missing (*n*)	1			0			1		
Race/ethnicity (*n* 580) %									
Non-Hispanic White	35·7			34·3			37·5		
Black/African American	8·6			8·3			9·0		
Hispanic/Latino	25·5			28·1			22·3		
Asian/Asian American	25·3			23·8			27·3		
Other or Unknown	4·8			5·6			3·9		
Mean age (n 580)		40·7	10·6		40·0	10·3		41·6	11.0
Mean total SSB consumption (oz./d) (*n* 580)		28·5	33·0		32·9	35·2		22·9	29·2
Mean anxiety score (*n* 570)		3·0	3·8		3·0	3·8		3·0	3·9
Missing (*n*)	10			7			3		
Mean BMI (*n* 578)		29·4	6·6		29·7	6·7		29·0	6·4
Missing (*n*)	2			1			1		

SSB, sugar-sweetened beverage.

Table 2 shows regression models predicting changes in consumption from anxiety during the pandemic. Across all participants, each point of anxiety score above the mean was associated with +0·65 oz./d change in consumption (Model 1). Because average consumption decreased across the whole sample, this suggested that participants with T2 anxiety greater than the sample mean experienced smaller declines in consumption than those at or below the sample mean. No significant demographic differences were found except for a gender difference in the control group (see online supplementary material, Supplemental 2).

**Table 2:** Regression models predicting change in SSB consumption (oz./d) from pandemic-era anxiety

	All participants (Model 1)			Sales ban (Model 2)			Control (Model 3)		
Variable	Coefficient	se	*P*	Coefficient	se	*P*	Coefficient	se	*P*
Anxiety score (at T2)	0·65	0·27	0·02	0·45	0·39	0·25	0·82	0·37	0·03
Condition (sales ban)	−2·33	2·34	0·32						
BMI at baseline	0·04	0·19	0·82	0·04	0·33	0·91	0·03	0·24	0·90
SSB consumption at T1	−0·66	0·04	0·00	−0·70	0·07	0·00	−0·63	0·05	0·00
Sex (male)	5·07	2·68	0·06	2·86	4·05	0·48	7·03	3·59	0·05
Race/Ethnicity (*v*. Non-Hispanic White)									
Black/African American	−0·91	4·75	0·85	11·25	7·49	0·14	−11·09	6·17	0·07
Hispanic/Latino	1·93	2·95	0·51	9·15	4·97	0·07	−3·12	3·69	0·40
Asian/Asian-American	0·09	3·02	0·98	1·65	4·42	0·71	−1·42	4·13	0·73
Other or Unknown	−3·65	5·96	0·54	−7·39	10·49	0·48	−3·45	7·22	0·63

SSB, sugar-sweetened beverage.

As models 2 and 3 show, the effects of anxiety on consumption were robust and statistically significant in the control group but not in the sales ban group. In the control group, a one-point above average anxiety score predicted a 0·82 oz./d (*P* = 0·03) change in consumption, compared with 0·45 oz./d change in the sales ban group (*P* = 0·25). As described above, because the average participant decreased consumption, these positive coefficients indicate smaller decreases for participants with more anxiety rather than increases in consumption.

**Fig. 1:**
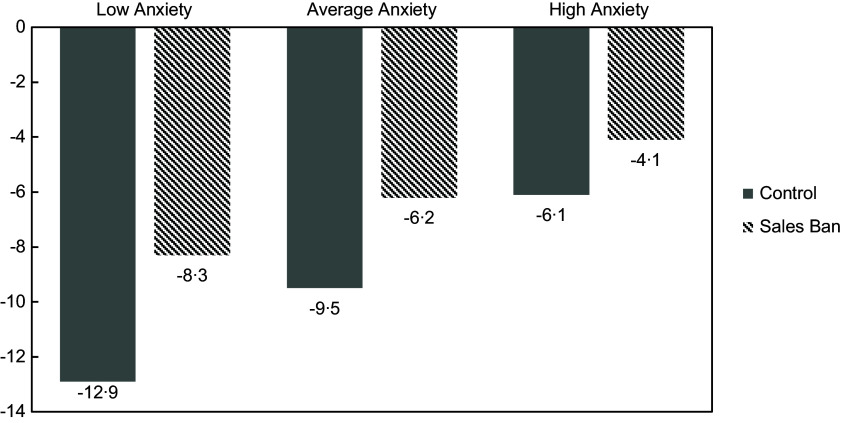
Predicted reduction in SSB consumption (oz./d) by anxiety level. Low, average, and high anxiety defined as 1 sd below the T2 mean, at the T2 mean, and 1 sd above the T2 mean. Regression models used to predict these average values (Table 2) were controlled for BMI, race/ethnicity, T1 consumption and sex. SSB, sugar-sweetened beverage.

To illustrate this, Fig. 1 displays *predicted* reductions in SSB for hypothetical average control group and sales ban participants at the T2 group anxiety score mean and at +/– 1 sd. At a control hospital, a participant with higher T2 anxiety would have an estimated 6·1 oz./d decrease in consumption, whereas a similar participant with lower anxiety would have a 12·9 oz./d decrease (Fig. 1). At a sales ban hospital, an average participant would reduce consumption by 4·1 oz./d at 1 sd above the T2 anxiety mean and 8·3 oz./d at 1 sd below, demonstrating smaller SSB reductions at higher levels of anxiety, but to a lesser extent than in the control group. The proportion of the difference between predicted anxiety values displayed in Fig. 1 appears similar across conditions, so we conducted sensitivity analyses with the outcome modelled as a percent reduction in SSB. Those analyses also suggested that anxiety had a larger impact on SSB change for the control group than the sales ban group. A test of interaction for condition and anxiety did not reach significance (see online supplementary material, Supplemental 3).

Finally, although the correlation between T1 and T2 anxiety was strong, (*r* = 0·48, *P* < 0·001), we calculated the same regression models as above, including change in anxiety from before (T1) to during the pandemic (T2) to determine how anxiety change related to consumption change (see online supplementary material, Supplemental 4). This analysis showed no statistically significant effects for change in anxiety. However, the pattern of coefficients for anxiety change (*β* = 0·58, *P* = 0·14 control *v*. *β* = 0·00, *P* = 0·99 sales ban) was similar to the pattern in Table 2 – wherein the control group’s coefficient for anxiety was larger than that for the sales ban group.

## Discussion

We extended an existing trial of workplace SSB sales bans^(1)^ into the COVID-19 pandemic. Given the stressors of the pandemic, we anticipated that anxiety would increase in our sample of hospital employees, which could lead to increases in SSB consumption. We further hypothesised that the reduced availability of SSB under a sales ban would buffer employees from stress-related increases in consumption.

First, as hypothesised, we found a significant increase in anxiety during the pandemic among hospital employees in this sample. We also found significant, positive, cross-sectional correlations between anxiety and consumption at T1 and T2, providing further evidence of a positive relationship between anxiety symptoms and SSB consumption.

Second, the pandemic was not associated with increases in consumption on average, likely due to this sample’s pre-existing trend of SSB decreases over time. However, we still observed important associations between anxiety during the pandemic and changes in consumption. Overall, hospital employees with higher anxiety scores during the pandemic experienced smaller reductions in consumption compared with those with lower anxiety scores. This relationship was statistically significant only for employees in the control group. The association between anxiety and change in consumption was weaker and non-significant in the sales ban group. This suggests that the sales ban protected individuals from anxiety-related changes in consumption. It seems plausible that anxiety-related consumption is more likely to be unplanned, unlike an employee choosing in advance to bring an SSB from home to drink on a meal break. Reduced availability of SSB under a sales ban might therefore prevent employees from engaging in impulsive consumption. Employees at control ban hospitals, however, had ready availability of SSB and showed some evidence of anxiety-related changes in consumption, which can generate detrimental health effects over time. For example, in the control group, a participant with higher anxiety (1 sd above the mean) would consume over 1240 more ounces of SSB/year than a comparable participant at the anxiety mean.

Although anxiety measured during the pandemic predicted change in SSB consumption, *changes* in anxiety did not have robust effects. There are multiple potential explanations for this. First, the timing of data collection (approximately two months after pandemic restrictions began locally) may have missed acute increases at the pandemic’s onset. Second, anxiety levels were relatively stable and strongly correlated within person from before to during the pandemic, with a mean change of only 1·2 points of anxiety score and *r* = 0·48, leaving limited variance in change scores to predict change in consumption. These factors may explain why an individual’s simple level of anxiety during the pandemic, which had more variability, was a better predictor of change in consumption.

Our study has several limitations. First, hospital employees likely had diverse experiences during the pandemic, such as amount of direct patient contact; we were unable to quantify this for analysis. There were also demographic differences between groups, which we attempted to account for in regression models. The sample size for this study was limited to that of the main trial; a larger sample size would have allowed for deeper exploration of differences in the anxiety–consumption relationship between control and sales ban groups, including potential demographic differences. We do not have data on what alternate strategies or behaviors might have been used to replace potential coping-motivated SSB consumption at work; an examination of that is part of our ongoing program of research. Compensatory consumption of sugar through foods or at home consumption of SSB is a possibility that must always be considered when examining reductions in the workplace context. However, pre-pandemic analyses from our larger trial found no evidence of either^(1)^. As noted in results, our outcomes could be modelled as a proportion of SSB change rather than change in oz./d. Because of a 12 oz./d SSB reduction, which should have health benefits, might represent 20 % of one participant’s consumption and 75 % of another’s, we chose to use oz./d as our outcome. Finally, although the control and sales ban groups were sampled during the same dates in 2019 and 2020, they were not at the same point in their participation in the main trial. Most sales bans had been in effect for over a year by mid-2019; therefore, some consumption change had already happened for most participants prior to T1, although the average sales ban participant was still consuming over 22 oz./d of SSB. Because of this difference in timing, this sub-study should be viewed not as a test of the effects of a sales ban on consumption, but rather a test of the effects of a sales ban on the anxiety–consumption relationship.

This study adds to the growing body of evidence supporting workplace SSB sales bans as an effective tool for promoting employee health. Our findings provide some evidence that, in addition to the previously established benefits of an SSB sales ban on health and well-being, this intervention could have the added benefit of protecting against SSB consumption that results from stress and anxiety.

## Supporting information

Jacobs et al. supplementary material Jacobs et al. supplementary material

## Supplementary material

For supplementary material accompanying this paper visit https://doi.org/10.1017/S1368980024000995
