# Supplementary material for: Did a workplace sugar-sweetened beverage sales ban reduce anxiety-related sugar-sweetened beverage consumption during the COVID-19 pandemic?
Source: Public Health Nutr. 2024 May 3;27(1):e139. doi: 10.1017/S1368980024000995 (PMC11374549; doi:10.1017/S1368980024000995)
Supplement: Jacobs et al. supplementary material [file S1368980024000995sup001.docx]

Supplement 1. Changes in anxiety and SSB consumption, T1 to T2

|  | All participants | | Control | | Sales Ban | |
| --- | --- | --- | --- | --- | --- | --- |
|  | Mean change (SD) | p | Mean change (SD) | p | Mean change (SD) | p |
| Anxiety score (N=454) | 1.2 (4.1) | .00 | 0.8 (3.9)^a^ | .00 | 1.7 (4.2)^a^ | .00 |
| SSB consumption (oz./day) (N=473) | -8.1 (31.7) | .00 | -9.5 (31.3)^b^ | .00 | -6.2 (32.1)^b^ | .00 |

p-values for between-group comparisons: ^a^ p<.001; ^b^ p=.26

Supplement 2. Sex differences

An unexpected finding was that men, particularly those in the control group, had a stronger anxiety-SSB change association than did women. As seen in the models displayed in Table 2 of the main paper, a man in the control group at the mean level of anxiety would be consuming 7 oz./day more SSBs at T2 than a woman with the same demographics and T1 SSB consumption. Initial levels of anxiety do not easily account for this finding; men in this sample had significantly lower anxiety scores at T1 compared to women (-0.9, SE 0.4, p<.05) and at T2 (-1.3, SE 0.5, p<.01), a commonly found sex difference. The small percentage of men in this sample hindered our ability to conduct further analyses, but in other COVID-era studies, men were less likely to report eating sweets to cope^(1)^ but drank more SSBs than women.^(2)^ Men may have been more likely than women to consume SSBs to cope with pandemic stressors. Further research should consider potential gender differences in stress-related consumption.

Supplement 3. Regression model predicting change in SSB consumption (oz./day) with interaction term for condition*anxiety

|  | All participants  (Model 1) | |
| --- | --- | --- |
| Variable | Coefficient (SE) | p |
| Anxiety score (at T2) | .38 | .08 |
| Condition (sales ban) | -.88 | .63 |
| BMI at baseline | .01 | .90 |
| SSB consumption at T1 | -.30 | .00 |
| Sex (male) | 1.50 | .33 |
| Race/Ethnicity (vs. Non-Hispanic White) |  |  |
| Black/African American | 1.23 | .65 |
| Hispanic/Latino | .75 | .66 |
| Asian/Asian-American | 2.24 | .19 |
| Other or Unknown | -2.46 | .47 |
| Condition*Anxiety score interaction | -.12 | .68 |

Supplement 4. Regression models predicting change in SSB consumption (oz./day) from change in anxiety scores

|  | All participants  (Model 1) | | Sales Ban  (Model 2) | | Control  (Model 3) | |
| --- | --- | --- | --- | --- | --- | --- |
| Variable | Coefficient (SE) | p | Coefficient (SE) | P | Coefficient (SE) | p |
| Change in anxiety score (T1 to T2) | .27 (.29) | .34 | .00 (.43) | .99 | .58 (.39) | .14 |
| Condition (sales ban) | -1.99 (2.37) | .40 |  |  |  |  |
| BMI at T1 | .06 (.19) | .75 | .01 (.33) | .97 | .08 (.24) | .73 |
| SSB consumption at T1 | -.66 (.04) | .00 | -.70 (.07) | .00 | -.61 (.05) | .00 |
| Sex (male) | 4.33 (2.70) | .11 | 2.35 (4.01) | .56 | 6.42 (3.66) | .08 |
| Race/Ethnicity (vs. Non-Hispanic White) |  |  |  |  |  |  |
| Black/African American | -1.70 (4.76) | .72 | 10.25 (7.48) | .17 | -12.09 (6.20) | .05 |
| Hispanic/Latino | 1.79 (3.01) | .55 | 8.32 (5.03) | .10 | -2.77 (3.78) | .46 |
| Asian/Asian-American | 0.23 (3.06) | .94 | 1.62 (4.47) | .72 | -.96 (4.20) | .82 |
| Other or Unknown | -3.91 (6.00) | .52 | -8.21 (10.48) | .43 | -3.50 (7.31) | .63 |

**SUPPLEMENT REFERENCES**

1. Prowse R, Sherratt F, Abizaid A, et al.. Coping With the COVID-19 Pandemic: Examining Gender Differences in Stress and Mental Health Among University Students. Front Psychiatry. 2021 Apr 7;12:650759. doi: 10.3389/fpsyt.2021.650759. PMID: 33897499; PMCID: PMC8058407.
2. Zhang Y, Tao S, Qu Y, et al.. Lifestyle behaviors and mental health during the coronavirus disease 2019 pandemic among college students: a web-based study. BMC Public Health. 2022 Nov 21;22(1):2140. doi: 10.1186/s12889-022-14598-4. Erratum in: BMC Public Health. 2023 Jun 22;23(1):1215. PMID: 36414957; PMCID: PMC9682808.
